# Progress of children with severe acute malnutrition in the malnutrition treatment centre rehabilitation program: evidence from a prospective study in Jharkhand, India

**DOI:** 10.1186/s12937-018-0378-2

**Published:** 2018-07-18

**Authors:** Anuraag Chaturvedi, Ashok K. Patwari, Deepa Soni, Shivam Pandey, Audrey Prost, Raj Kumar Gope, Jyoti Sharma, Prasanta Tripathy

**Affiliations:** 1 Public Health Foundation of India, Gurugram, India; 2 Hamdard Institute of Medical Sciences and Research and H.A.H.Centenary Hospital, Hamdard University, New Delhi, India; 3 Indian Institute of Public Health, Gurugram, India; 4 University College London, London, UK; 5 Ekjut, Chakradharpur, Jharkhand, India

**Keywords:** Wasting, Assessment of nutritional status, Severe acute malnutrition, Malnutrition treatment centers, Food and nutrient intake, India

## Abstract

**Background:**

In Jharkhand, Malnutrition Treatment Centres (MTCs) have been established to provide care to children with severe acute malnutrition (SAM). The study examined the effects of facility- and community based care provided as part the MTC program on children with severe acute malnutrition.

**Method:**

A cohort of 150 children were enrolled and interviewed by trained investigators at admission, discharge, and after two months on the completion of the community-based phase of the MTC program. Trained investigators collected data on diet, morbidity, anthropometry, and utilization of health and nutrition services.

**Results:**

We found no deaths among children attending the MTC program. Recovery was poor, and the majority of children demonstrated poor weight gain, with severe wasting and underweight reported in 52 and 83% of the children respectively at the completion of the community-based phase of the MTC program. The average weight gain in the MTC facility (3.8 ± 5.9 g/kg body weight/d) and after discharge (0.6 ± 2.1 g/kg body weight/d) was below recommended standards. 67% of the children consumed food that met less than 50% of the recommended energy and protein requirement. Children experienced high number of illness episodes after discharge: 68% children had coughs and cold, 40% had fever and 35% had diarrhoea. Multiple morbidities were common: 50% of children had two or more episodes of illness. Caregiver’s exposure to MTC’s health and nutrition education sessions and meetings with frontline workers did not improve feeding practices at home. The take-home ration amount distributed to children through the supplementary food program was inadequate to achieve growth benefits.

**Conclusions:**

Recovery of children during and after the MTC program was suboptimal. This highlights the need for additional support to strengthen MTC program so that effective care to children can be provided.

## Background

Wasting in children is the result of recent rapid weight loss or failure to gain weight due to hunger and/or disease. Children suffering from wasting have low immunity, are susceptible to developmental delays and face an increased risk of death [1]. The prevalence of severe wasting (WHZ less than − 3 of the median WHZ in WHO Child Growth Standards) in the state of Jharkhand (located in Eastern India) is high and greater than the national average: in 2016, 11.4% of children aged 0–35 months were severely wasted in Jharkhand, compared to a national average of 7.5%. Therefore, at any point of time, around 0.6 million children in the state are severely wasted [2].

To address this challenge, Jharkhand’s State government has established 88 malnutrition treatment centres (MTCs) through the National Health Mission (NHM), where children with severe acute malnutrition (SAM)—severe wasting and/or mid-upper arm circumference (MUAC) < 115 mm and/or bilateral pitting oedema— receive medical and therapeutic care based on the WHO and Indian Academy of Pediatrics protocols [3–5]. Detection of children with SAM in the community is done by the Anganwadi workers (AWWs) of the Integrated Child Development Services (ICDS) program. Once at the MTC, children are again screened for SAM and admitted to MTC if they meet the admission criterion. After admission, children are treated for their medical complications and fed locally prepared therapeutic formula F-75 every 2 h for 2 days, and subsequently, F-100 six time daily for 48 h.^1^ After completing 4 days at the MTC, children are alternately fed F-100 and locally prepared semi-solid therapeutic food until the child is discharged from the centre. During stabilization phase, nurses conduct health and nutrition education sessions for caregivers on child feeding, illness management, immunization and hygiene. Children are discharged from MTC when they have (a) no signs of bilateral pitting oedema, fever and/or infection, and (b) gained at least 5 g/kg body weight/d. After discharge, children are transitioned to the community phase of the program, where they are followed up in the community by frontline workers; enrolled into the ICDS supplementary nutrition program; and returned for three follow-up visits to the MTC every 15 days during the 2 months following discharge.

Between July 2009–June 2011, 3595 children were treated in the 48 MTCs of Jharkhand [6]. MTC results highlight the following challenges: high defaulting rates; poor weight gain once children are back in the community; and low attendance rate in the follow-up visits. However, there is limited about the reasons for this persistent challenges [6]. Current literature on MTC, or Nutrition rehabilitation centers (NRCs) as they are called in other parts of India, have mainly reported treatment outcomes for the facility-based phase of the program. During the community-based phase however, children often return to an environment where optimal care for children remains a challenge. The objective of our study is to describe children’s outcomes for both phases of the program, determine access and utilization of post-discharge services, and measure compliance of health and nutrition practices advised by MTC in order to inform future implementation of the MTC and broader nutrition programs in India.

## Methods

### Study sites and participants

We randomly selected 14 out of the 48 MTCs operating before March 2011 for the study. A total of 150 children admitted to these MTCs between December 20, 2011 and January 31, 2012 were enrolled and followed at three points: at admission (first contact), discharge (second contact), and after completion of 2 months of community phase of the program (third contact). We interviewed 116 children and their caregivers at discharge (34 defaulted and left the MTC against the advice of the MTC staff), and 100 children after completion of community phase of the program (18 had migrated and 2 could not be located).

### Data collection

We obtained data on the health and nutritional status of children at admission and discharge from MTC records. Caregivers (mothers) were interviewed by local trained investigators after obtaining their written informed consent. In case respondents could not read or write, alternate witnessed consent process was followed. We pretested the study instruments in the community and collected data on: (a) mothers’ socio-economic and demographic characteristics; (b) children’s morbidity during first and third contact (c) dietary intake in the past 24 h during third contact; (d) utilisation of Integrated child development services(ICDS) and health services during first and third contact; (e) knowledge, attitude and practice of caregivers on child feeding during all three conatcts; (f) anthropometric measurements at all three contacts.

All anthropometric assessments were conducted as per WHO (2006) protocol. Anthropometric measurements were conducted by trained field investigators. Children were weighed without clothes on portable electronic scales accurate to the nearest 0.1 kg that were calibrated daily. Portable infantometers and stadiometers accurate to 0.1 cm were used to record length and height respectively. Mid upper-arm circumference was measured using MUAC tapes supplied by UNICEF. Anthropometric measurements were taken twice and the mean value was recorded.

Dietary information was obtained using 24-h dietary recall by probing caregivers in detail. Using standardized kitchen utensils, cut outs and kitchen weighing scales, investigators recorded the food amount, ingredients and feeding time. For any reported outside snacks, samples of packaged local snacks were collected for measurement reference. Knowledge of caregivers on MTC recommended child feeding practices was assessed through structured open ended questions and categorized as right or wrong. The feeding practices were evaluated from children’s 24-h dietary recall survey.

### Data analysis

Statistical analysis was performed using SPSS, version 19 (SPSS Inc., Chicago). We used Anthro software, WHO version 3.0.1, to generate anthropometric indices. Diet analysis was conducted by trained nutritionists. We converted dietary data into individual raw food weights and analyzed these using Diet Soft version 1.1.9. The diet’s energy and protein nutrient adequacy was compared against recommendations for catch up growth (at least 150 kcal/kg/day and 4 g protein/kg/day). [7] Analysis of variance (ANOVA) and chi-square tests were used to test for statistical significance and changes in anthropometric indices were tested with repeated measure ANOVA tests.

Poisson regression was used to identify factors associated with follow-up visits to MTC, and McNemar’s test was applied to analyze change in child feeding knowledge of caregivers after admission in the MTC program.

## Results

Children admitted in MTC were very young (Table 1); 85% of children were in the 6–23 months’ age group. Over half (54%) of children were girls, and 68% belonged to scheduled tribe(ethnic/tribal) families. The majority of caregivers were 18–25 years’ old and had received no formal schooling. Enrollment characteristics—socio-demographic and wasting status—of children who defaulted were similar to those who completed the 2 months’ follow-up.

**Table 1:** Characteristics of children and their mothers in the MTC program

	First Contact (Admission)	Second contact (Discharge)	Third Contact (Community- based phase)	Defaulted & Loss to follow-up
Children’s Sex (%)				
Male	46	46.2	47	44
Female	54	53.8	53	56
Children’s Age, months (%)				
6–11	38.7	33.9	25	32
12–23	45.3	49.2	55	46
24–35	12	14.4	14	16
36+	4	2.5	6	6
Mother’s religion(%)				
Hindu	29.3	30.5	30	28
Muslim	6	5.1	6	4
Sarna	54	56.8	55	60
Others	10.7	7.6	9	8
Mother’s caste(%)				
SC	9.3	11	10	10
ST	68	70.3	71	72
OBC	15.3	14.4	14	12
General	7.4	4.2	5	6
Mother’s age (%)				
18–25	46.7	47.5	47	42
26–30	24	22.9	24	26
31+	29.3	29.7	29	32
Mother’s education (%)				
No schooling	67.3	66.1	66	70
1–8	26.1	29	29	28
9+	6.6	5.2	5	2
Total children and mothers	150	116	100	50

### Child growth

Tables 2 describes the anthropometric indices of children and their growth status at discharge and follow up. Out of 150 children admitted, 116 (77.3%) children completed their treatment at the MTC, their average length of stay at MTC was 16.2(SD: ±4.9) days. About 22% of children achieved the recommended weight gain of 8 g/kg/day in MTC, with the average weight gain being 3.8 g/kg/day (SD: ±5.9). WHZ and WAZ scores improved significantly between admission and discharge, though all children remained in the severe underweight category (WAZ < − 3) at discharge. After transition of the children to community-based phase of the program, their average weight gain per day reduced to 0.6 g/kg/day (SD: ±2.1) and many became undernourished again. At follow-up, more children were severely undernourished than at discharge; 52% of children were severely wasted (mean WHZ: − 3.1 ± 1.5) and 83% were underweight (mean WAZ: − 4.0 ± 1.1) at follow-up.

**Table 2:** Anthropometric indices of children and weight gain during facility-based and community-based phase of the MTC program

	Facility based Phase			Community–based phase *N* = 100	*p*-value
	Admission *N* = 150	Discharge *N* = 116	*p*-value		
WHZ (mean ± SD)	−3.2 ± 1.9	−2.8 ± 1.1	0.001	−3.1 ± 1.5	0.018^ǂ^
Severe (%)	54	40		52	
Moderate (%)	46	38		27	
Normal (%)	–	22		21	
WAZ (mean ± SD)	−4.2 ± 1.2	−3.8 ± 1.1	< 0.001	−4.0 ± 1.1	0.001^ǂ^
Severe (%)	84	71		83	
Moderate (%)	16	26		14	
Normal (%)	–	3		3	
Average weight gain/body weight/day (grams)		3.8 ± 5.9		0.6 ± 2.1	< 0.001^ǂ^

^ǂ^Significant difference between community-based phase and discharge

### Dietary intake by children

Table 3 describes the mean macronutrient and micronutrient intake of children as well as the nutrition adequacy of their diet during community phase of the program. The recommendations for adequate energy and protein intake for children are at least 150 Kcal/kg bodyweight/day, and 4 g/Kg bodyweight/day, respectively. 67 and 63% children consumed food that met less than 50% energy and protein requirement on the day preceding the survey. We found no significant association between the children’s age or sex and energy and protein adequacy levels. The energy and protein intake of severely wasted children was significantly lower compared to that of moderately wasted children and normal children. The mean iron and zinc intake in the previous day’s diet was inferior to recommended daily allowances for all age groups. Mean meal frequency was similar among severely wasted (2.4 ± 2.0), moderately wasted (2.8 ± 2.1) and normal children (3.5 ± 2.7).

**Table 3:** Mean macro and micronutrient intake of children after discharge by age group and growth status, and nutrient adequacy from home diet on the day preceding the survey

	Energy intake (mean Kcal ± SD)	*P*-value	Protein intake (mean g ± SD)	*P*-value	Iron intake (mean mg ± SD)	*P*-value	Zinc intake (mean mg ± SD)	*P*-value
Age group								
6–11 months	266.8 ± 135.3		7.7 ± 4.7		1.4 ± 0.9		0.9 ± 0.5	
12–23 months	459.7 ± 211.8	< 0.001^1^	12.0 ± 6.5	0.05	2.5 ± 1.9	0.01^1^	1.4 ± 0.8	0.01^1^
≥ 24 months	484.0 ± 163.1		12.4 ± 7.4		3.6 ± 4.2		1.6 ± 0.6	
WHZ status								
Severe	365.1 ± 198.9		9.1 ± 6.1		2.6 ± 3.0		1.3 ± 0.7	
Moderate	465.3 ± 168.1	0.01^1^	11.7 ± 5.7	0.05	2.2 ± 2.1	0.8	1.3 ± 0.7	0.4
Normal	504.0 ± 221.9		13.9 ± 6.8		2.6 ± 1.7		1.6 ± 0.8	
Nutrient adequacy (%)^2^								
< 50%	67%		63%					
50–66%	22%		18%					
66–100%	11%		19%					

^1^Significant difference between groups; ANOVA, ^2^Energy adequacy: subject’s calorie intake/(150 Kcal/kg bodyweight); Protein: subject’s protein intake/(4 g/kg bodyweight)

### Morbidity

We assessed morbidity among children in last 2 months preceding the survey (results not shown). Caregivers reported episodes of cold or cough in 68% of children (median episode:1; average duration 6.6 ± 3.8 days), followed by fever in 40% children (median episodes:1.5; average duration 4.5 ± 2.1 days) and diarrhoea in 35% children (median episode:1; average duration 4.8 ± 3.2 days). Multiple morbidities were common in children after discharge from MTCs: About 15% children reported all three morbidities; while 26% reported two morbidities during last 2o months preceding the survey. Few children remained free from illness: 21% children reported no illness. Highest reported morbidities were found in the 12–23 months’ age group: 74% cough and cold incidences, 38% diarrhoea, and 34% fever were reported from this age group. No significant association was observed between illness and gender.

### Follow up visits to MTCs

Sixteen children were lost to follow-up due to migration in the community-based phase of the study. Out of the 100 children, 19% attended all three follow-up visits to MTC. 19% of children attended two and 21% children attended one follow-up visits. 41% of children did not attend any follow-up visit. The Poisson regression analysis after adjusting socio-demographic factors, mother’s education, growth status at discharge, child morbidities and length of stay at MTC, indicated that increased number of follow-up visits to MTC was significantly (*p* < 0.05) associated with awareness about the need for three follow-up visits (adjusted PR: 4.3; 95%CI: 2.0–9.1); caregivers being accompanied by someone to MTC (adjusted PR: 2.1; 95% CI: 1.4–3.1); and children having MUAC< 115 mm at discharge (adjusted PR: 1.9; 95% CI: 1.1–3.6).

### Knowledge and practice about child feeding and hygiene practices

We measured and analyzed caregivers’ knowledge on child feeding and hygiene at three time intervals: at admission; at discharge; and again during community phase to assess retention of MTC delivered messages (Table 4). McNemar’s test determined that compared to pre-admission knowledge, more caregivers had correct knowledge on diet modification at discharge (21% vs 52%; *p* < 0.001) and food quantity (1% vs 35% *P* < 0.001), while no difference was observed for knowledge on feeding frequency and food consistency. Caregivers retained this knowledge during the community phase; the proportion of caregivers with correct knowledge on child feeding practices was similar at discharge and in the community-based phase. We examined children’s diet in the previous day before the survey in the community-based phase; this showed a gap between caregiver’s knowledge and practice; 37% caregivers knew that children should be fed five or more times per day, but only 14% of the caregivers practiced this.

**Table 4:** Caregivers correct knowledge and practice on child feeding and hygiene at three time intervals

	At Admission	At discharge	*P*-value^1^	At follow-up	*P*-value^2^
Knowledge about feeding frequency (%)	30	34	0.60	37	0.75
Gave at least five meals to the child in last 24 h (%)	–	–	–	14	–
Knowledge about dietary modification (%)	21	52	< 0.001	56	0.6
Modified child’s food in last 24 h (%)	–	–		0	–
Knowledge about food consistency (%)	53	57	0.5	60	0.7
Knowledge about food quantity (%)	2	35	< 0.001	33	0.8
Awareness of six MTC promoted hygiene practices	–	2.8 ± 1.8		4.4 ± 2.1	< 0.001

^1^Difference between admission and discharge, ^2^Difference between follow-up and discharge

### Linkages with local nutrition and health services after discharge

Jharkhand’s Integrated Child Development Services (ICDS) programme mandates free distribution of double amount of take-home ration (THR) of rice, daal (lentils), soyabean, oil and sugar to all children after their discharge from MTC. The majority of caregivers received less than one-third of the entitled THR (Table 5). The majority of caregivers reported contact by frontline workers after discharge from MTC—60% Anganwadi workers (AWW) and 54% ASHA (Sahiya) contacted caregivers two or more times in last 2 months preceding the survey. About 36% caregivers reported monthly weight measurement of children, while 31% said that child had not been weighed (Table 5). Consumption of Iron supplementation by children was reported by 26% caregivers and deworming by 15% caregivers after discharge from MTC.

**Table 5:** Linkages between caregivers and frontline workers and services received in the community-based phase of the program

Distribution of Take Home Ration (THR) in the previous month (%)	≤ 1/3 amount	1/3–2/3 amount	Full amount	Mean amount (grams ± SD)
Rice (3000 g)	81	19	–	1129 ± 362.5
Lentils (750 g)	78	22	–	269.4 ± 92.5
Soyabean (500 g)	63	37	–	171.1 ± 94.6
Cooking oil (250 g)	16	60	24	123.5 ± 66.1
Sugar (1900 g)	100	–	–	249.1 ± 104.5
Contact with Frontline workers in last 2 months (%)	None	One contact	Two contacts	Three or more contacts
AWW	12	28	38	22
ASHA (Sahiya)	21	25	33	21
ANM	36	29	28	7
Weight measurement by frontline workers in last two months (%)	Not weighed	Weighed once	Weighed twice	Weighed thrice
	31	33	26	10

## Discussion

This study examines children’s progress in the MTC program—in facility, and after discharge in the community-based phase of the program. Our result show, children gained weight below the national standards of care, suffered multiple morbidities, defaulted on follow-up visits, and received food supplements that were inadequate to meet their needs. These are important findings and strengthens the need for finding appropriate solutions to provide effective care for SAM children in the program.

MTC or NRC program in India has shown high survival outcomes (< 5% child deaths) [6, 8] and a similar performance was observed in our study. In spite of many children being severely wasted at discharge, we found no deaths among children in the program. A study in rural India that followed 409 severely wasted children reported low case-fatality (1.2–2.7%) among children without community management of acute malnutrition [9].

Recovery among children remained poor as majority of the children demonstrated poor weight gain during both phases of the program. The children’s average weight gain in the MTC facility and after discharge was below the recommended national and international standards [10, 11], and lower than that achieved by other programs in India [12] and Community Based Management of Acute Malnutrition (CMAM) programs elsewhere [13]. The poor weight gain suggests of sub-optimal quality of the therapeutic food in MTC, and low energy and protein dense foods at home. In our study, two out every three children received less than 50% of the energy required (at least 150 Kcal/kg body weight/day) and protein required (4 g/Kg body weight/day) from their previous day’s home diet. Consumption of high energy density meals is known to result in higher weight gains, and when small meals are consumed, their energy density becomes even more important [14, 15]. Dietary analysis of children highlighted inadequacy of energy-and-nutrient dense foods in their meals. Caregivers fed foods of thin consistency, minimal diversity, and fed small portion in main meals. In our study, caregivers typically fed children 2–3 meals (rice with lentils or vegetable gravy) in a day, and provided occasional snacks (biscuits, local savories and *murhi*-rice puffs) between meals. While we did not assess household food security(HHFS), this has been shown to affect diet, nutrition and infant feeding practices. Better HHFS status has been reported to be associated with the type of complementary foods given to the infants [16], and was also observed as a significant predictor of receiving a minimally acceptable diet for infants [17].

Morbidity was common post-discharge, about 50% of all children reported more than two illnesses in the 2 months of the community phase of the program. Micronutrient deficiencies could have led to morbidity by impairing immune function. We found that children’s diet was essentially vegetarian and micronutrient intake (iron, zin and calcium) remained inferior to recommended daily allowances for all age groups. In the 2 months after discharge from MTC facility, no micronutrient supplements were provided except vitamin A under routine immunization program. Diarrhoea remains a common health problem after treatment of SAM [18, 19] which is unsurprising given that children return to the same environment characterised by inadequate food, poor access to sanitation and safe water. Khanum and Ashworth observed that 92% children after treatment of SAM in Bangladesh experienced diarrhoea (mean:7episodes; range 0–30) during 1 year and weight gain tended to be lower in children who experienced more episodes of diarrhoea. [20]

Our results showed no difference in the weight gain of children who did not attend any follow-up visit compared to those who visited MTC after discharge. Caregivers defaulted on their follow-up visit to MTC due to low awareness about the need for re-visits and non-availability of family members or acquaintance to accompany caregivers to MTC. The low attendance rate for follow-up visits was similar to a previous study in Jharkhand where 62.4% of the 2770 discharge children did not return for any follow-up visits, and only 14.9% children completed the three follow-up visits. Caregiver’s awareness about the services and their opportunity cost are important factors as they may influence the health seeking behavior. Puett and Guerrero [21] in a study in Pakistan and Ethiopia observed that caregiver’s low awareness about the services, distance to the facility and the accompanying transportation cost negatively impacted the access to CMAM services. Role of frontline workers has shown to be central in reducing default rates. In the neighboring state of Bihar, involvement of ASHA contributed to the sharp decline in default rate to < 20%. [22] In our study, frontline workers played a major role in the identification and admission of SAM children to MTCs but became inactive during the follow-up visits: ASHA and AWWs identified 51 and 40% SAM cases; accompanied caregivers to MTC for admission in 44 and 31% cases; but in follow-up visits accompanied caregivers only in 13 and 5% cases respectively. Effective actions are required to improve compliance to the follow-up visits. Improved community messaging regarding follow-up visits and integration of follow-up visit with existing incentive based health delivery services of health workers could improve follow-up visits of caregivers to MTC.

Health and nutrition education sessions conducted by nurses at MTC showed mixed results. Caregiver’s awareness about the child feeding practices improved for few topics, but many still remained unaware about the correct feeding practices at the time of discharge. The result underscores the need for support to strengthen the content and delivery of MTC education sessions. Our results also showed that majority caregivers did not adopt optimal feeding practices known to them. In-depth interviews (data not reported) with caregivers showed feeding frequent meals remained a challenge due to competing demands of siblings; workload in the house and field; and limited cooking resources at home. Discrepancies between maternal knowledge and practice had been found to be related with psychological and social factors that included cultural experiences, existing norms and expectations, available support, time constraints and work load. [23] We also observed that the regular contact between caregivers and frontline workers did not improve the child feeding practices at home. A capacity assessment study of AWWs in Gujarat observed that AWWs had adequate knowledge on infant young child feeding practices but could not engage effectively with caregivers, lacked the skills to assess child’s breastfeeding and complementary feeding practices and provided general advice to caregivers irrespective of child’s condition [24]. As observed elsewhere, frontline workers consider weight measurement in the growth monitoring sessions as an end in itself, and fail to discuss with caregivers the reasons for growth faltering and solutions to address it [25, 26]. A literature search of successful community-based rehabilitation programs for the period from 1980 to 2005 found involvement of motivated and trained staff who taught caregivers about child feeding and health promotion through structured program; had sessions that required caregivers to practice what they had learned; and provided continuous reinforcement to caregivers through home visits. [7] Sub-optimal child feeding practices of caregivers despite their regular contact with frontline workers calls for strengthening the counseling capacity of frontline ICDS and NHM workers so that caregivers could be guided for compliance to the MTC promoted feeding and hygiene practices.

All severely underweight children and those discharged from MTCs are entitled for double THR amount. That distribution of THR amount was inadequate for the children to realize growth benefits. Previous studies on ICDS have attributed poor coverage of take-home ration to low demand for the product due to poor ration quality; distribution to non-eligible households; and insufficient stocks at AWCs [27, 28]. Intra-household sharing of ration targeted at the children remains a challenge for food based programs. Results from our previous study showed that out of the reduced THR received by severely underweight children, 70% was further shared with other family members [29]. For better targeting of children in the community-based phase, the existing ICDS provision of hot cooked meals to 3–6 years’ age children can be modified and extended to children with SAM so to provide them with cooked meals comprising of essential macro and micronutrients.

Our study had several limitations: (a) Fifty children had defaulted after admission to MTC and could not be traced. Mortality among defaulted children remains unknown. Although no differences existed between children who defaulted and those who continued in the program, the study instruments might not have captured all the differences; (b) Morbidity information is subject to uncertainty as it was not validated, and caregivers might have failed to accurately recall illness episodes due to 2 month’s recall period. (c) Breastmilk volume consumed by children was not assessed, the inclusion of which would have improved the reported nutrient intake among the children (d) About three-fourth of the study children belonged to the tribal families, caution must be taken to generalize the findings to SAM children treated at rehabilitation centres in general. (e) Data on complicated and non-complicated SAM at MTC was not available, analysis of which could have provided more insights about the children’s recovery in the program.

## Conclusions

To date facility- based management remains the accepted strategy to treat children with SAM in India. The following suggestions could be summarized from this study to improve the performance of MTC program.

1. Provide appropriate therapeutic foods in MTC to ensure minimum average weight gain. At home, aim to provide high-energy, high-protein diet to children for rapid weight gain
2. Set up a responsive system at community level to provide health services during morbidity episodes. Provide micronutrient supplementation after discharge to improve immune function
3. Invest in improving the technical and counseling skills of MTC nurses and frontline workers so that active support can be extended to caregivers on child feeding and health care practices.
4. Modify and extend hot cooked meals served to 3–6 years’ age children under ICDS supplementation program to children with SAM so to provide them with meals comprising of essential macro and micronutrients

## Abbreviations

ANM: Auxiliary Nurse Midwife
ASHA: Accredited Social Health Activist
AWW: Anganwadi Workers
CMAM: community based management of acute malnutrition
HAZ: Height for age z score
ICDS: Integrated Child Development Services
MTC: Malnutrition Treatment Centre
NHM: National Health Mission
SAM: Severe Acute Malnutrition
THR: Take Home Ration
WAZ: Weight for age Z score
WHZ: weight for height z score

## Footnotes

1 Composition of locally prepared F-75(per 1000 ml): cow’s milk or toned milk (300 ml), sugar (60 g), vegetable oil (25 g) puffed rice (25 g), water (to complete 1000 ml). Composition of F-100 (per 1000 ml): cow’s milk or toned milk (750 ml), sugar (25 g), vegetable oil (20 g), puffed rice (70 g), water (to complete 1000 ml). Composition of locally prepared semi-solid therapeutic food (per 100 g): roasted groundnuts (25.5 g), milk powder (30.6 g), coconut oil (15.3 g) and sugar (28.6 g)
